# Alteration of brain connectivity in neurologically asymptomatic patients with chronic kidney disease

**DOI:** 10.1097/MD.0000000000025633

**Published:** 2021-04-23

**Authors:** Yoo Jin Lee, Eunjae Yoon, Sihyung Park, Yang Wook Kim, Si Eun Kim, Junghae Ko, Jin Han Park, Kang Min Park, Il Hwan Kim, Bong Soo Park

**Affiliations:** a Department of Internal Medicine; b Department of Neurology, Inje University Haeundae Paik Hospital, Busan, Korea.

**Keywords:** brain, cognition, kidney failure, network

## Abstract

Our previous study demonstrated that patients with end-stage renal disease had decreased structural and functional brain connectivity, and there was a significant association between brain connectivity and cognitive function. The aim of this study was to evaluate the alterations of structural and functional connectivity using graph theoretical analysis in neurologically asymptomatic patients with relatively early-stage chronic kidney disease (CKD).

We enrolled 18 neurologically asymptomatic patients with early CKD and 28 healthy controls. All the subjects underwent diffusion-tension imaging and resting functional magnetic resonance imaging. We calculated structural and functional connectivity based on diffusion-tension imaging and resting functional magnetic resonance imaging using a graph theoretical analysis. Then, we investigated differences of structural and functional connectivity between the CKD patients and the healthy controls.

All the measures of structural connectivity were significantly different between the patients with CKD and healthy controls. The global efficiency, local efficiency, mean clustering coefficient, and small-worldness index were decreased, whereas the characteristic path length was increased in the patients with CKD compared with healthy controls. The structural betweenness centrality of the left calcarine and right posterior cingulum was also significantly different from that in healthy participants. However, all the measures of global functional connectivity in patients with CKD were not different from those in healthy controls. In patients with CKD, the functional betweenness centrality of the right insular cortex, right occipital pole, and right thalamus was significantly different from that in healthy participants.

There are significant alterations of the global structural connectivity between the patients with CKD and the healthy subjects, whereas the global functional connectivity of the brain network is preserved. We find that the efficiency of the structural brain network is decreased in the patients with CKD.

## 1 Introduction

Chronic kidney disease (CKD) is defined as abnormalities of kidney structure or function that last for more than 3 months.^[1]^ A condition is diagnosed as CKD if there is a decrease in the glomerular filtration rate (GFR, GFR < 60 mL/min/1.73 m^2^), presence of structural abnormalities detected by imaging, or abnormalities in urine tests such as proteinuria.^[1]^ Patients with CKD have many complications and have higher mortality rates than the general population. Cardiovascular complications are the most common type of complication and an important cause of death among patients with CKD.^[1]^ Neurological complications, such as peripheral polyneuropathy, autonomic dysfunction, stroke, uremic encephalopathy, and cognitive impairment (CI), are also common and important complications in patients with CKD.^[2]^ Many previous studies with cross-sectional and longitudinal designs have shown that CI is more common in patients with CKD compared to the general population.^[3]^ CI is inversely related to renal function.^[4]^ As a result, reduced kidney function, regardless of whether caused by CKD or acute kidney injury, results in decreased brain function, which causes CI.^[5]^

Currently, the study of brain connectivity and its relation to cognition is an emerging field in neuroscience. Brain connectivity is crucial to elucidating how neurons and neural networks process information. Many neuroimaging studies have identified alterations in the brain network related to CI in various neurological disorders.^[6,7]^ Graph theoretical analysis is among the common methods for investigating brain connectivity.^[8]^ It is a branch of mathematics concerned with how networks can be encoded and their properties measured. It has an advantage in that it simplifies complex brain connectivity into a simple model to improve clinical interpretability.^[8]^ A previous study using graph theoretical analysis in healthy participants revealed a significant correlation between the intelligence quotient (IQ) and network measures derived from graph theoretical analysis, such as the mean clustering coefficient, global efficiency, local efficiency, small-worldness index, and characteristic path length, thus indicating that brain connectivity is related to cognitive function.^[9]^

In the graph theory, global efficiency represents the efficiency of information transfer from one region to the whole network, and it is computed as the average nodal efficiency of all nodes.^[8,10]^ The local efficiency defines the efficiency of information transfer from each region to the neighboring regions, and the local efficiency of a network is conventionally defined as the average of the local efficiencies of all nodes.^[8,10]^ The characteristic path length is defined as the average number of edges in the shortest path between all pairs of nodes. The small-worldness index is related to network efficiency for brain networks and relies on the global transitivity of the network and its average shortest path length. The global efficiency and characteristic path length reflect the integration of the brain network, while local efficiency indicates the degree of segregation in the brain network.^[8,10]^

Recent studies performing quantitative analyses of the brain have reported reduced total cortical and subcortical volumes, which are associated with CI in patients with end-stage renal disease (ESRD).^[11]^ In addition, patients with ESRD have lesser cortical thickness than healthy controls, especially in the frontal cortex.^[12,13]^ Other studies using a tract-based spatial statistical analysis of diffusion tensor imaging (DTI) to investigate the microstructural changes in the white matter over the whole brain in patients with ESRD have revealed lower fractional anisotropy (FA) and higher mean diffusivity in the widespread white matter in patients with ESRD than in healthy controls.^[14,15]^ There have been several studies on brain morphology and connectivity in patients with ESRD using variable magnetic resonance (MR) sequences, such as DTI, voxel-based volumetry based on T1-weighted images, MR spectroscopy, and arterial spin-labeling MR perfusion imaging.^[11,12,16–20]^

There are relatively sufficient studies on brain network changes in patients with ESRD. However, there are few studies on changes in the brain network in patients with stage 3 CKD. The aim of this study was to evaluate the alterations of structural and functional connectivity using graph theoretical analysis based on DTI and resting state functional MRI (rs-fMRI) in neurologically asymptomatic patients with relatively early-stage CKD compared to healthy controls.

## 2 Materials and method

### 2.1 Subjects

This study was approved by the institutional review board and was prospectively performed in a single tertiary hospital. We enrolled 18 neurologically asymptomatic patients with early CKD from May 2019 to December 2019. The patients were defined as those that with stage 3 CKD: GFR between 30 and 59, and no previous history of neurological or psychiatric disorders. GFR was calculated using the Chronic Kidney Disease Epidemiology Collaboration equation. We excluded the patients with structural lesions on their brain MRI.

We also enrolled an age- and gender-matched control group of 28 healthy participants without any prior significant past medical, neurological, or psychiatric history. Both the patients with CKD and healthy controls had normal brain MRI findings on visual inspection.

### 2.2 Brain MRI

All participants underwent MRI using the same imaging protocol. All scans were performed using a 3.0T MRI scanner equipped with a 32-channel head coil (AchievaTx, Phillips Healthcare, Best, The Netherlands). Scans obtained included sagittal-oriented 3-dimensional T2- and T1-weighted images and coronal-oriented 3-dimensional fluid-attenuated inversion recovery images to evaluate structural lesions in the participants’ brains. Moreover, all participants underwent DTI and rs-fMRI that was suitable for graph theoretical analysis. DTI was performed using spin-echo single shot echo-planar pulse sequences with a total of 32 different diffusion directions (TR/TE = 8620/85 ms, FA = 90°, slice thickness = 2.25 mm, acquisition matrix = 120 × 120, FOV = 240 × 240 mm^2^, and *b*-value = 1000 s/mm^2^). The rs-fMRI was performed using multislice echo-planar imaging sequences (TR/TE = 3000/30 ms, FA = 65°, slice thickness = 4.4 mm, acquisition matrix = 128 × 128, FOV = 220 × 220 mm^2^, scan time = 7 min 30 s).

### 2.3 Image processing and analysis

We performed most of the DTI analysis to evaluate structural connectivity using the DSI studio (http://dsi-studio.labsolver.org). The procedures for the graph theoretical analysis were as follows. The first step was to create a tractography from the DTI data, which included reading and parsing DICOM files, reconstructing to characterize the major diffusion direction of the fiber, and fiber tracking. The next step was to generate a connectivity matrix, which was calculated using the count of the connecting tracts. The Automated Anatomical Labeling template was used for brain parcellation, and every white matter fiber was evaluated for extreme points. This step included obtaining a whole-brain fiber track, placing seeding regions in the whole brain, spatial normalization, definition of the region of interest, and creating a connectivity matrix. Last, we calculated the graph theoretical network measures from the connectivity matrix.

The rs-fMRI data were analyzed using Statistical Parametric Mapping software packages (SPM, version 12, Functional Imaging Laboratories, London, UK), as well as the functional connectivity toolbox, CONN (Cognitive and Affective Neuroscience Laboratory, Massachusetts Institute of Technology, Cambridge, MA), running under MATLAB (MathWorks, Sherborn, MA). The rs-fMRI data were preprocessed using the standard spatial preprocessing steps of realignment, slice-time correction, coregistration, normalization in the Montreal Neurological Institute space, and smoothing with a 6-mm Gaussian kernel. Functional connectivity analysis was then performed using the CONN toolbox (version 17).

Using data from the structural and functional connectivity matrices based on DTI and rs-fMRI, we calculated the global network measures including global efficiency, local efficiency, mean clustering coefficient, characteristic path length, and small-worldness index. In addition, we obtained the measure of betweenness centrality to investigate the local network topology.

### 2.4 Statistical analysis

Comparisons of clinical characteristics and network measures were conducted using the chi-squared test for categorical variables and the Student *t* test or Mann–Whitney test for numerical variables. We also conducted the correlation analysis between the global network measures and clinical characteristics with Spearman test. Categorical variables were presented in terms of both frequency and percentage. Numerical variables were presented as mean ± standard deviation. A *P* value of less than 0.05 was considered to indicate statistical significance for all calculations. All the statistical tests were performed using MedCalc (MedCalc Software version 18.6, Ostend, Belgium).

## 3 Results

### 3.1 Demographic and laboratory characteristics of the participants

Eighteen patients with CKD stage 3 were enrolled. The mean estimated glomerular filtration rate (eGFR) was 39.68 ± 9.37 mL/min/1.73 m^2^. Eight of 18 patients (44.4%) had underlying diabetes and 13 patients (72.2%) had hypertension. The male-to-female ratio was 1:1. Other demographic and laboratory characteristics were described in Table 1.

**Table 1:** Demographic and clinical characteristics in patients.

	Patient	Control	
Variables	Mean with SD	Mean with SD	*P* value
Age (by year)	65.89 ± 9.87	65.00 ± 6.35	.701
Gender (N, % female)	9/18, 50%	18/28, 64.3%	.373
Hemoglobin (g/dL)	11.88 ± 1.89	13.46 ± 1.39	.030
Hematocrit (%)	35.67 ± 5.47	40.64 ± 3.38	.020
Protein (g/dL)	7.17 ± 0.66	6.94 ± 0.40	.336
Albumin (g/dL)	4.02 ± 0.37	4.07 ± 0.35	.716
Aspatate aminotransferase (U/L)	22.39 ± 6.55	25.93 ± 5.38	.119
Alanine aminotrasferase (U/L)	18.78 ± 8.23	23.31 ± 13.46	.268
BUN (mg/dL)	26.01 ± 6.35	16.57 ± 3.56	<.001
Creatinine (mg/dL)	1.68 ± 0.36	0.89 ± 0.12	<.001
Estimate glomerular filtration rate (eGFR) (mL/min/1.73 m^2^)	39.68 ± 9.37	73.26 ± 10.87	<.001
Sodium (mmol/L)	140.89 ± 2.56	141.08 ± 2.14	.870
Potassium (mmol/L)	4.72 ± 0.45	4.24 ± 0.27	.003
Chloride (mmol/L)	106.16 ± 3.97	104.54 ± 1.98	.211
Calcium (mg/dL)	8.40 ± 1.23	8.84 ± 0.31	.154
Phosphate (mg/dL)	3.62 ± 0.50	3.60 ± 0.55	.966
Total CO_2_ contents (mmol/L)	23.86 ± 3.02	26.90 ± 2.49	.034

### 3.2 Structural connectivity

Measures of structural global topology were significantly different between the patients with CKD and healthy participants. All the measures of global structural connectivity (global efficiency, local efficiency, mean clustering coefficient, characteristic path length, and small-worldness index) were significantly different between the patients with CKD and healthy controls (Table 2). The global efficiency, local efficiency, mean clustering coefficient, and small-worldness index were decreased, whereas the characteristic path length was increased in the patients with CKD compared to healthy controls.

**Table 2:** Measures of structural global topology in patients with chronic kidney disease and healthy subjects.

	Patients with CKD		Healthy controls				
Variables	Mean	SD	Mean	SD	Difference	95% CI	*P* value
Global efficiency	0.9282	0.0844	1.5253	0.1360	−0.5971	0.5301– 0.6641	<.001
Local efficiency	1.1675	0.1819	2.4513	2.8653	−1.2839	1.1415– 1.4262	<.001
Mean clustering coefficient	0.1132	0.0461	0.2483	0.0874	−0.1351	0.0943– 0.1760	<.001
Characteristic path length	3.9069	0.4005	4.2500	0.4048	−0.3431	0.0942 –0.5920	.008
Small-worldness index	0.0801	0.0352	0.2678	0.0903	−0.1877	−0.0194 to –0.0152	<.001

**Figure 1:**
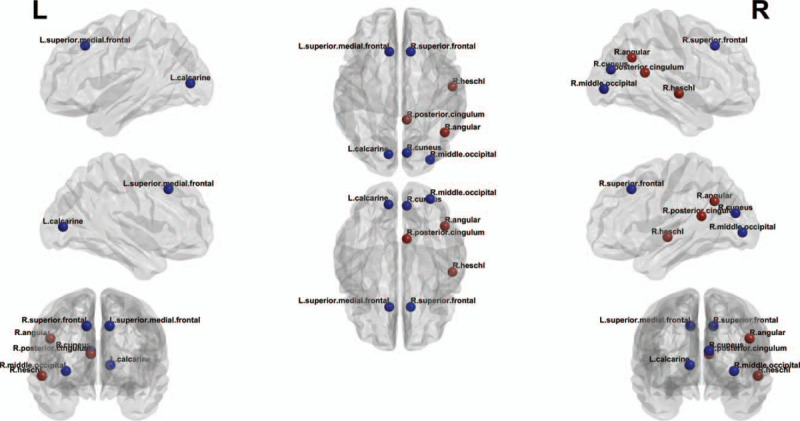
Differences in local structural connectivity between the chronic kidney disease patients and healthy subjects. It shows that there are many regions with alterations in the local structural connectivity in the ESRD patients. Red circles indicate the nodes with increased betweenness centrality, whereas blue circles represent the nodes with decreased betweenness centrality in the chronic kidney disease. ESRD = end-stage renal disease.

There was also a significant difference in the measures of local structural connectivity between the 2 groups. The betweenness centrality of the right superior frontal gyrus, left superior medial frontal gyrus, left calcarine gyrus, right angular gyrus, right posterior cingulum, right cuneus, right Heschl's gyrus, and right middle occipital gyrus in the patients with CKD was significantly different from that in healthy participants (Fig. 1).

### 3.3 Functional connectivity

Measures of functional global topology were not significantly different between the patients with CKD and healthy participants. All the measures of global functional connectivity (global efficiency, local efficiency, mean clustering coefficient, characteristic path length, and small-worldness index) were not significantly different between the patients with CKD and the healthy controls (Table 3).

**Table 3:** Measures of functional global topology in patients with chronic kidney disease and healthy subjects.

	Patients with CKD		Healthy controls				
Variable	Mean	SD	Mean	SD	Difference	95% CI	*P* value
Global efficiency	0.5066	0.0253	0.5028	0.0149	0.0038	−0.0096 to 0.0162	.543
Local efficiency	0.7193	0.0305	0.7287	0.0195	−0.0094	−0.009 to 0.0162	.224
Mean clustering coefficient	0.5006	0.0507	0.5199	0.0309	−0.0193	−0.0445 to 0.0059	.129
Characteristic path length	2.2415	0.1032	2.2684	0.0703	−0.0269	−0.0802 to 0.0265	.314
Small-worldness index	0.2229	0.0150	0.2291	0.0010	−0.0061	−0.0138 to 0.0014	.109

**Figure 2:**
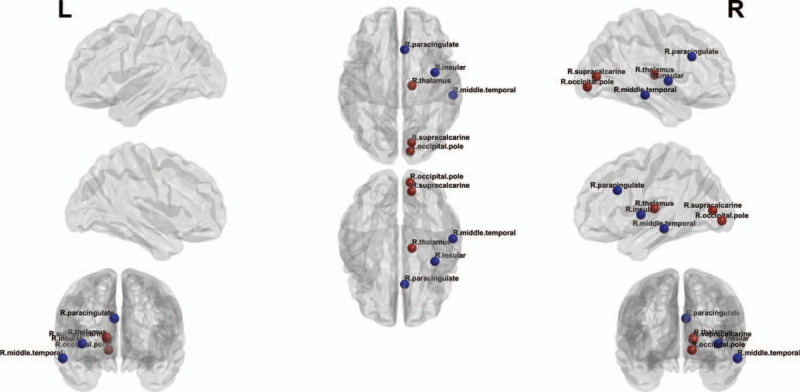
Differences of local functional connectivity between the chronic kidney disease patients and healthy subjects. It shows that there are many regions with alterations in the local functional connectivity in the ESRD patients. Red circles indicate the nodes with increased betweenness centrality, whereas blue circles represent the nodes with decreased betweenness centrality in the chronic kidney disease patients.

However, there was a significant difference in the measures of local functional connectivity between the 2 groups. The betweenness centrality of the right insula, right paracingulate gyrus, right occipital pole, right supracalcarine gyrus, right thalamus, and right middle temporal gyrus in the patients with CKD was significantly different from that in healthy participants (Fig. 2).

### 3.4 Correlation analysis

The correlation analysis found that there was no significant correlation between the measures of global structural and functional connectivity and the clinical characteristics, including hemoglobin, albumin, BUN, creatinine, estimate glomerular filtration rate, and total CO_2_ contents. (see Table, supplemental Table 1 and supplemental Table 2 which illustrates the correlation analysis between the global structural and functional network measures and clinical characteristics).

## 4 Discussion

The main findings of this study are that there is an alteration of global structural brain connectivity in patients with relatively early CKD; however, the global functional connectivity is preserved. Surprisingly, all the parameters (global efficiency, local efficiency, mean clustering coefficient, characteristic path length, and small-worldness index) representing the global structural connectivity were significantly different from that in the healthy controls, whereas all the parameters representing the functional connectivity were not significantly different from that in the healthy controls. Patients with CKD had significant alterations in the network hubs compared with the healthy controls. In addition, the regions where alterations in local connectivity were observed were significantly different between the patients with CKD and healthy participants. The results can be interpreted that neurologically asymptomatic patients with early CKD have alterations of structural connectivity. However, clinical neurologic symptoms do not develop because the functional connectivity is preserved.

In general, structural connectivity is an anatomical concept of connectivity that refers to the existence and structural integrity of tracts that connect different brain areas, whereas functional connectivity refers to the statistical dependence of the signal from different areas and reveals the functionally integrated relationship between spatially separated brain regions.^[21]^ Functional connectivity is not always consistent with structural connectivity. Whether structural or functional connectivity is important and what causes dissociations between structural and functional connectivity is not clearly determined. However, several previous studies have demonstrated that clinical presentations of many neuropsychiatric diseases, especially schizophrenia, are less correlated with structural connectivity of the brain and more strongly correlated with functional connectivity, including behavioral performance and emotional measures.^[22,23]^ Furthermore, a study in patients with spastic diplegic cerebral palsy showed that the motor network had significantly lower efficiency of functional connectivity compared with the intact structural connectivity and lower structure-function coupling than the control group.^[24]^ All these studies suggested that functional connectivity, rather than structural connectivity, closely reflects clinical manifestations in the various neuropsychiatric diseases. In several previous studies, including our previous study, patients with ESRD showed alterations of structural and functional connectivity compared with the general population.^[13,19,25]^ However, in patients with early CKD, there is structural alteration of brain connectivity but no alteration of functional connectivity. Alterations in structural connectivity have various causes, but functional connectivity is maintained by various compensatory mechanisms in patients with early CKD. This study was conducted in clinically asymptomatic patients with CKD; the preserved functional connectivity seems to better reflect the clinical manifestation. It can be assumed that the alteration of structural connectivity develops in patients with CKD and the alteration of functional connectivity eventually deteriorates in patients with ESRD, which result in neurological abnormalities.

Although the global functional connectivity in patients with CKD was not different from that in healthy controls, there were several regions with significant differences in the local functional connectivity. We investigated the local structural and functional connectivity with the measure of betweenness centrality. The betweenness centrality quantifies the number of times a node acts as a bridge along the shortest path between 2 other nodes, and hubs can be defined as nodes with a number of links that greatly exceeds the average. Therefore, the betweenness centrality can represent the extent to which a node plays important role in a network and it reveals hubs of the brain network. Thus, we can assume that there were alterations of hubs of functional connectivity in the patients with CKD compared with healthy controls, although the global functional connectivity was preserved.

Factors affecting alterations in brain connectivity in patients with ESRD are varied, including nephrogenic factors such as uremic toxins, anemia, vascular calcification and chronic inflammation, as well as factors related to treatment such as intradialytic hypotension, which can cause cerebral ischemia.^[26]^ Of these, the most important factor influencing brain connectivity in patients with renal disease, including those with ESRD, is thought to be the accumulation of uremic toxins. The elimination of uremic toxins can result in a significant improvement in brain connectivity. This can be confirmed by comparing brain connectivity before and after kidney transplantation or before and after initiation of dialysis.

A recent study showed that cognitive function and brain connectivity in patients with ESRD can be improved after successful kidney transplant surgery, although the degree and pattern of recovery differed depending on the affected region of the brain.^[27]^ It is speculated that improved cognitive function performance after kidney transplantation may be associated with the successful removal of toxins.^[28]^ Moreover, kidney transplantation is associated with restoration of a normal biochemical milieu, maintenance of hemodynamic stability, and the removal of dialytic stress, which may be beneficial for cognition improvement. Kidney transplantation restores the role of the kidney as an endocrine organ, which includes functions such as regulating anemia by secreting erythropoietin and regulating blood pressure through the renin angiotensin aldosterone system, as well as removing waste products. Therefore, further studies are needed to identify the improvement in brain connectivity and neurologic manifestations due to the elimination of uremic toxins as a result of dialysis, which mainly consists of removing uremic waste products.

Although uremic toxins and other factors play important roles in the alteration of brain connectivity in patients with ESRD, it is not yet clear why there is an alteration of structural connectivity and preserved functional connectivity in patients with stage 3 CKD. However, patients with stage 3 CKD also share common risk factors and are on the same disease spectrum as patients with ESRD, and therefore, the factors affecting brain connectivity will not be significantly different.

There were multiple strengths associated with our study. This is the first study to investigate the differences of structural and functional connectivity in patients with early CKD compared with a healthy population. Moreover, we conducted DTI and rs-fMRI simultaneously to analyze the structural and functional brain connectivity in patients with early CKD.

However, there were several limitations to this study. First, the size of the data set was small because we conducted a small-scale, single-center study. It could be possible that our results represent false positive due to small sample size. Thus, we additionally calculated the power of this study using the Power and Sample Size Program (http://ps-power-and-sample-sizecalculation.software.informer.com/download/). It revealed that the statistical power of this study was sufficient to exclude type 1 error (all of them had more than 80%). Second, owing to the cross-sectional design of the current study, we only observed the connectivity in patients with CKD at 1 time point. Longitudinal studies will provide information on alterations in brain connectivity as kidney function worsens. Third, we could not exclude the effects of several comorbidities associated with CKD such as anemia, hypertension, and diabetes. This is especially important for the functional connectivity based on rs-fMRI, as these factors may affect blood oxygen level-dependent signals.^[29]^ However, it was difficult to enroll the patients with CKD without hypertension or diabetes. Finally, we did not perform detailed neuropsychological tests to assess the correlation between the measures of brain connectivity and cognitive function.

To overcome these limitations, large-scale studies are needed. Moreover, the effect of underlying disease may be excluded by analyzing the brain connectivity of patients with CKD caused by the same underlying disease. Further research will also be needed on alterations of brain connectivity in patients with acute kidney injury and the changes in brain connectivity resulting from the recovery of acute kidney injury.

## 5 Conclusion

There are significant alterations of the global structural connectivity between the patients with CKD and the healthy subjects, whereas the global functional connectivity of the brain network is preserved. We find that the efficiency of the structural brain network is decreased in the patients with CKD.

## Author contributions

**Conceptualization:** Jin Han Park, Il Hwan Kim.

**Data curation:** Sihyung Park, Yang Wook Kim, Si Eun Kim.

**Formal analysis:** Junghae Ko, Kang Min Park.

**Writing – original draft:** Yoo Jin Lee, Eunjae Yoon.

**Writing – review & editing:** Bong Soo Park.

## Supplementary Material

Supplemental Digital Content
