# Supplementary material for: Alteration of brain connectivity in neurologically asymptomatic patients with chronic kidney disease
Source: Medicine (Baltimore). 2021 Apr 23;100(16):e25633. doi: 10.1097/MD.0000000000025633 (PMC8078245; doi:10.1097/MD.0000000000025633)
Supplement: Supplemental Digital Content [file medi-100-e25633-s001.docx]

**Supplementary table 1**. Correlation analysis between the global structural network measures and clinical characteristics

| Variable | Clustering coefficient average weighted | | | Global efficiency weighted | | | Local efficiency weighted | | Network characteristic path length weighted | | | Small-worldness weighted | | |
| --- | --- | --- | --- | --- | --- | --- | --- | --- | --- | --- | --- | --- | --- | --- |
|  | *R* | *p*-value | *R* | | *p*-value | *R* | | *p*-value | | *R* | *p*-value | | *R* | *p*-value |
| Albumin (g/dl) | -0.061 | .810 | 0.069 | | .784 | 0.077 | | .762 | | 0.277 | .265 | | 0.006 | .980 |
| BUN (mg/dl) | 0.220 | .380 | -0.281 | | .258 | -0.332 | | .177 | | -0.364 | .137 | | 0.099 | .695 |
| Creatinine (mg/dl) | -0.013 | .957 | -0.249 | | .318 | -0.380 | | .120 | | -0.257 | .302 | | -0.162 | .519 |
| eGFR (mL/min/1.73 m^2^) | -0.102 | .686 | 0.082 | | .747 | 0.174 | | .488 | | 0.212 | .399 | | 0.009 | .970 |
| Hemoglobin (g/dl) | -0.059 | .816 | -0.224 | | .371 | -0.300 | | .227 | | 0.247 | .323 | | -0.054 | .832 |
| Total CO_2_ (mmol/L) | -0.023 | .928 | -0.062 | | .807 | -0.199 | | .427 | | 0.155 | .539 | | 0.011 | .964 |

R : Correlation coefficient, BUN : Blood Urea Nitrogen, eGFR : estimated Glomerular Filtration Rate

**Supplementary table 2**. Correlation analysis between the global functional network measures and clinical characteristics

| Variable | Average path length | | | Clustering coefficient | | | Global efficiency | | Local efficiency | | | Small-worldness | | |
| --- | --- | --- | --- | --- | --- | --- | --- | --- | --- | --- | --- | --- | --- | --- |
|  | *R* | *p*-value | *R* | | *p*-value | *R* | | *p*-value | | *R* | *p*-value | | *R* | *p*-value |
| Albumin (g/dl) | 0.301 | .224 | 0.260 | | .296 | -0.258 | | .301 | | 0.326 | .186 | | 0.259 | .298 |
| BUN (mg/dl) | -0.072 | .775 | 0.008 | | .974 | 0.004 | | .987 | | 0.124 | .624 | | 0.138 | .584 |
| Creatinine (mg/dl) | -0.295 | .235 | -0.164 | | .514 | 0.214 | | .393 | | 0.044 | .860 | | -0.011 | .964 |
| eGFR (mL/min/1.73 m^2^) | 0.044 | .253 | 0.137 | | .587 | -0.212 | | .399 | | 0.018 | .944 | | 0.001 | .996 |
| Hemoglobin (g/dl) | 0.044 | .861 | -0.041 | | .870 | -0.045 | | .857 | | 0.112 | .659 | | -0.090 | .722 |
| Total CO_2_ (mmol/L) | 0.292 | .239 | 0.093 | | .713 | -0.219 | | .382 | | 0.026 | .919 | | -0.112 | .659 |

R : Correlation coefficient, BUN : Blood Urea Nitrogen, eGFR : estimated Glomerular Filtration Rate
